# Early clinical features of new-onset refractory status epilepticus (NORSE) in adults

**DOI:** 10.1186/s12883-022-03028-y

**Published:** 2022-12-20

**Authors:** Anna Haanpää, Sini M. Laakso, Antti Kinnunen, Leena Kämppi, Nina Forss

**Affiliations:** 1 Department of Neurology, Neurocenter, Helsinki University Hospital, PB 372, 00029 HUS Helsinki, Finland; 2 Department of Clinical Neurosciences, University of Helsinki, PB 22, 00014 University of Helsinki Helsinki, Finland; 3 Department of Clinical Neurophysiology, Helsinki University Hospital, PB 340, 00029 HUS Helsinki, Finland; 4 Epilepsia Helsinki, Department of Neurology, Neurocenter, Helsinki University Hospital, PB 372, 00029 HUS Helsinki, Finland

**Keywords:** FIRES, Epilepsy, Encephalitis, Intensive care, Neuronal antibodies

## Abstract

**Background:**

The aim of this study was to identify early clinical features of patients with new-onset refractory status epilepticus (NORSE) that could direct the treatment in the first days of hospitalisation.

**Methods:**

A retrospective cohort study of adult NORSE patients treated in the intensive care units of Helsinki University Hospital 2007-2018.

**Results:**

We found 19 adult NORSE patients who divided into three subgroups on the basis of their clinical features: viral encephalitis (*n* = 5, 26%), febrile infection-related epilepsy syndrome (FIRES) (*n* = 6, 32%) and afebrile NORSE (*n* = 8, 42%). FIRES and afebrile NORSE patients remained without confirmed etiology, but retrospectively two paraneoplastic and two neurodegenerative causes were suspected in the afebrile NORSE group.

Viral encephalitis patients were median 64 years old (IQR 55-64), and four (80%) had prodromal fever and abnormal findings in the first brain imaging. FIRES patients were median 21 years old (IQR 19-24), all febrile and had normal brain imaging at onset. In the afebrile NORSE group, median age was 67 (IQR 59-71) and 50% had prodromal cognitive or psychiatric symptoms. FIRES patients differed from other NORSE patients by younger age (*p* = 0.001), respiratory prodromal symptoms (*p* = 0.004), normal brain MRI (*p* = 0.044) and lack of comorbidities (*p* = 0.011). They needed more antiseizure medications (*p* = 0.001) and anesthetics (*p* = 0.002), had a longer hospital stay (*p* = 0.017) and more complications (*p* < 0.001).

**Conclusions:**

Among febrile NORSE patients, FIRES group was distinctive due to patients’ young age, prodromal respiratory symptoms and normal first brain imaging. These features should be confirmed by subsequent studies as basis for selecting patients for early intensive immunotherapy.

**Supplementary Information:**

The online version contains supplementary material available at 10.1186/s12883-022-03028-y.

## Background

New-onset refractory status epilepticus (NORSE) is a rare but potentially life-threatening neurological condition, in which a person develops status epilepticus without apparent cause [1]. The condition was first described in the 1960s by pediatric neurologist Lyon [2]. In 2018, a multinational panel of epilepsy specialists published a consensus definition for NORSE and its subcategory FIRES (Febrile infection-related epilepsy syndrome) [1]. NORSE was defined as a clinical presentation of new-onset refractory status epilepticus (RSE) without structural, toxic, or metabolic cause, prior epilepsy, or other relevant neurological disorder [1]. In FIRES, a febrile infection precedes RSE, with fever starting between 2 weeks and 24 hours prior to onset of status epilepticus [1]. Identified etiologies behind NORSE are heterogeneous, including viral encephalitis, autoimmune encephalitis, and paraneoplastic and neurodegenerative diseases [3]. Despite extensive diagnostic workup, over half of the patients remain without a confirmed etiology [3].

Due to heterogenous and often cryptogenic etiology, rarity of the condition and lack of systematic studies, there is no consensus on the optimal treatment of NORSE. Conventional antiseizure medications (ASMs) and anesthetics have shown limited success. Untargeted immunotherapy is commonly used although the evidence for its benefits for all NORSE patients is inconclusive [4]. In recent years, dysfunction of the innate immune system has been identified as a possible pathogenic mechanism of FIRES, and promising studies with interleukin antagonists have been reported for this subgroup [5, 6].

NORSE challenges the whole neurocritical team as the condition requires extensive diagnostic work-up whilst managing the treatment-resistant RSE. Diagnostic tests may take days or weeks to complete, but as longer duration of RSE is associated with higher mortality [7], there is a need for rapid identification of the patients that would benefit from antiviral or immunological therapies.

In this retrospective study, we set out to systematically explore the early clinical symptoms and findings in patients with NORSE to find features that would help to direct the treatment in the very first days of hospitalisation.

## Methods

### Study design and setting

This is a retrospective cohort study of adult NORSE patients treated in Helsinki University Hospital (HUS) between 1st January 2007 and 31st December 2018.

HUS provides neurological emergency services and intensive care treatment 24 hours a day for a hospital district of 1.7 million inhabitants. In addition, HUS offers demanding specialized medical care services across hospital district boundaries for a catchment area of 2.2 million inhabitants. According to local treatment guidelines, patients with super-refractory status epilepticus (SRSE) not responding to anesthetic treatment are referred to HUS from the whole catchment area.

### Definitions

NORSE and FIRES were defined according to the definitions set by an international panel of epilepsy specialists in 2018 [1]. RSE was defined as persisting status epilepticus despite administration of at least one appropriately dosed parenteral benzodiazepine and another intravenous ASM, and super-refractory status epilepticus (SRSE) as SE persisting ≥24 hours after onset of anesthesia and recurring during appropriate anesthesia or after withdrawal of anesthesia and requiring anesthetic reintroduction [1].

### Patient selection

We identified all SE patients over 16 years of age treated in the intensive care unit of HUS between 2007 and 2018 from the electronic patient database based on ICD-10 code G41 (SE).

**Fig. 1:**
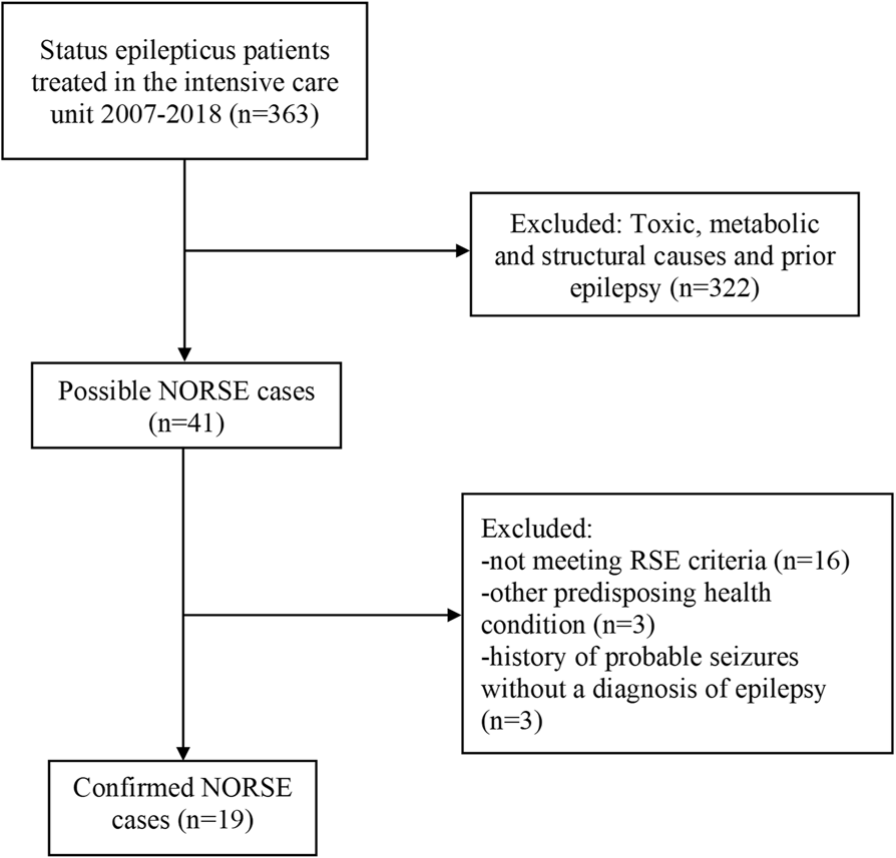
Identifying the study cohort of NORSE

We found 363 SE patients and reviewed their medical records to find patients who fulfilled the NORSE criteria [1]. We excluded 322 patients with an obvious cause for SE and obtained 41 possible NORSE cases that were evaluated in detail by the research group. Another 22 patients were excluded for not meeting the criteria for RSE, having predisposing conditions or recent history of seizures. Finally, 19 patients with NORSE were identified and included in the study. A flow chart of the patient selection is presented in Fig. 1.

### Data collection

A trained medical doctor (A.H) collected the data, including demographics, prodromal symptoms starting two weeks before the onset of SE, diagnostic studies, treatments used and their duration, complications, Status epilepticus severity score (STESS) [8] and outcome.

### Measures

The first brain MRI performed and CSF sample taken were used in the analysis. CSF leukocytes > 5 × 10^6^/l were regarded as abnormal. Reference values for CSF proteins were 200-500 mg/l in patients ≤50 years and 250-650 mg/l in patients > 50 years.

EEG reports from the first seven days after admisson to hospital were included. EEG reports included analysis of 30-minutes full-scale and 8-channel recordings and continuous 8-channel recordings used in clinical judgement. Ictal EEG-findings in this study were classified as focal, multifocal, or generalized based on EEG reports. EEG source data evaluation was made by an experienced clinical neurophysiologist (A.K.) in six cases as reports were missing from the electronic patient database due to software changes over time. All patients had either EEG reports or EEG source data available. Predominant seizure type was selected if several seizure types were present.

Complications were categorized according to Complication Burden Index (CBI) [9] into 13 classes: respiratory system (respiratory dysfunction, need for mechanical ventilation), infections, cardiovascular system (need for vasopressors, EF < 50%, heart rate < 50/min), hypo/hyperglycemia (requiring medical intervention), renal system (insufficiency defined as creatinine 100 μmol/l or GFR below the normal age-related reference value), electrolytes/acid-base balance (defined as pH < 7,2, S-Sodium < 130 mmol/l or > 150 mmol/l or other disturbance requiring medical intervention), psychiatric (delusions, paranoia, depression), coagulation system (thromboembolism, Hb < 80 g/l or need for blood products), nervous system (neuropathy, brain oedema, stroke), liver (fP-Nh4-ion > 50 μmol/l or P-Bil > 20 μmol/l or a five-fold increase in normal age-related ALAT level), skin/allergic reactions (rash, decubitus), musculoskeletal (rhabdomyolysis, fractures, dislocations), and gastrointestinal (melena, ileus).

ASMs included all antiepileptic medications administered intravenously or perorally. Used anesthetics were propofol, thiopental, ketamine and midazolam. Antimicrobial medication consisted of aciclovir combined with ceftriaxone and/or doxicyclin. Immunotherapies included three days high-dose intravenous methylprednisolone continued with peroral prednisolone, intravenous immunoglobulin treatment, plasma exchange, azathioprine, rituximab and/or allopregnalone.

General SE guidelines were followed in SE treatment [9]. It is noteworthy that in Finland all units in the Emergency medical services (EMS) are trained to identify a potential SE and start first-line medication. Physician- and nurse-based units are dispatched to high-risk SE calls, and they are able to start second-line treatment (from 2016 onwards) and induce anesthesia and intubate the patient already at the scene. No specific diagnostic or treatment protocol of NORSE was used during the study period in HUS, and therefore autoimmune encephalitis or paraneoplastic antibody panels were not routinely tested from all patients. Instead, various sets of antibodies were screened. Towards the end of the study period screening became more systematic. Detailed data on antibody testing is given in supplement tables (Tables S1-S3).

### Outcome

Outcome was measured by defining mRS at discharge from the tertiary hospital and at one year after the onset of NORSE. If the patient did not have a documented assessment by a physician one year after the hospitalization, mRS was defined at the nearest timepoint to one year. mRS scores were categorized in three classes: good (mRS 0-3), poor (mRS 4-5), and death (mRS 6). Need for permanent ASM and mortality at the end of the follow-up were also determined. Follow-up extended from onset of NORSE until the last health care contact before the end of data collection (31st December, 2020).

### Statistical analysis

Results are displayed as number and percentage or median and interquartile range (IQR). The Mann-Whitney U and Kruskal-Wallis tests were used for continuous variables and the Fisher’s exact test for categorical variables. Binary logistic regression was used to search for explanatory factors for good or poor outcomes and for use of long-term ASM. *P*-values < 0.05 were considered significant and two tailed tests were used. Statistical analyses were performed using SPSS software (version 26, IBM Corp, NY, USA).

## Results

Out of the 363 SE patients treated in the intensive care unit, 19 (5%) fulfilled the criteria for NORSE. Median age was 55 years (IQR 27-65) and 58% were female. Seventeen (90%) of patients were white, one was black African descent and one Syrian arab. The total mortality in the cohort was 16% (*n* = 3) at discharge, 32% (*n* = 6) one year after NORSE episode and 47% (*n* = 9) in the end of the follow-up in December 2020.

### Subgroups of NORSE

NORSE patients could be categorized into three subgroups based on their febrile status and found etiology. Febrile patients included patients with either a laboratory confirmed viral encephalitis (*n* = 5, 26% of all patients) or patients fulfilling the diagnostic criteria of FIRES (*n* = 6, 32% of all patients). Patients without fever were termed afebrile NORSE (*n* = 8, 42% of all patients). FIRES and afebrile NORSE patients (74% of all patients) remained without a confirmed etiology.

Out of the viral encephalitis patients, four (80%) tested positive for Herpes simplex virus 1 (HSV-1) and one (20%) for tick-borne encephalitis (TBE) from the CSF. Infectious etiology was confirmed within 3-12 days from symptom onset, whereas FIRES and afebrile NORSE groups went through diagnostic workup continuing throughout the intensive care treatment without confirmative results.

Two patients in the FIRES subgroup had abnormal antibody findings, which, however, were found undiagnostic: One had mildly elevated serum thyroid peroxidase (TPO) antibodies but the clinical features were not typical of Hashimoto’s encephalitis and one had mild elevation of voltage-gated kalium channel complex antibodies (VGKC) in CSF and serum but LGI1 (Leucine-rich glioma-inactivated 1) and CASPR2 (Contactin-associated protein-like 2) antibodies were normal in both, leaving the role of the finding undetermined. This patients also had a weak signal of paraneoplastic antibodies, SRY-Box Transcription Factor 1 (SOX-1) and Zinc finger protein 4 (Zic4), detected in the CSF.

**Fig. 2:**
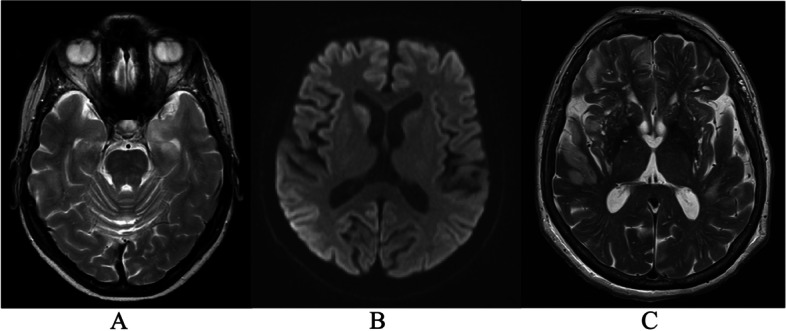
Abnormal MRI findings in the afebrile NORSE group (*n* = 3). **A**) Hyperintensity in mesial parts of the left temporal lobe (T2 sequences; patient 12 in supplemental table S3). **B**) Restricted diffusion in right nucleus caudatus and putamen and marginally in the cortex of the right hemisphere (DWI sequences, patient 16 in supplemental table S3). **C**) Bilateral hyperintensity of white and grey matter in temporal and frontal lobes extending to the parietal lobe (T2 sequences, patient 17 in supplemental table S3)

Additionally, a retrospective review revealed possible etiologies for four cases in the afebrile NORSE group. In two patients, a paraneoplastic syndrome could be suspected based on later diagnosis of a malignancy: one patient died of metastasized breast cancer eight months after NORSE and one patient of metastasized lung cancer 28 months after NORSE. Neither of these patients had been tested for paraneoplastic antibodies during the NORSE episode. In two cases, findings suggestive of a neurodegenerative disease emerged: One patient had CSF 13-4-4 marker answered positive 2 weeks after the acute NORSE episode, and the rapidly developed dementia as well as brain MRI suggestive for prion disease (Fig. 2, image B). One patient was diagnosed with frontotemporal dementia 16 months after NORSE based on repeated neuropsychological evaluation and CSF biomarkers. None of these underlying conditions were identified during the NORSE period and therefore could not be confirmed to be causative for NORSE.

The description of NORSE subgroups is presented in Table 1 and given treatments and features of intensive care in Table 2. Individual patient data is presented in greater detail in supplementary material including comorbidities, laboratory tests, EEG, brain imaging findings, given immunotherapies and mRS scores at discharge (Tables S1-S3).

**Table 1:** Demographics, prodromal symptoms and diagnostics in the identified NORSE subgroups

	All patients (***n*** = 19)	FIRES (***n*** = 6)	Viral encephalitis (***n*** = 5)	Afebrile NORSE group (***n*** = 8)	FIRES vs non-FIRES
**Demographics**					
Female	11 (58%)	3 (50%)	2 (40%)	6 (75%)	NS
Age at onset	55 (27-65)	21 (19-24)	64 (55-64)	67 (59-71)	*p* = 0.001
**Comorbidities**	10 (53%)	0	3 (60%)	6 (75%)	*p* = 0.011
**Prodromal symptoms**					
fever	10 (53%)	6 (100%)	4 (80%)	0	NS
headache	5 (26%)	2 (33%)	3 (60%)	0	NS
respiratory symptoms	4 (21%)	4 (67%)	0	0	*p* = 0.004
gastrointestinal symptoms	4 (21%)	2 (33%)	2 (40%)	0	NS
cognitive symptoms	5 (26%)	0	1 (20%)	4 (50%)	NS
psychiatric symptoms	1 (5%)	0	0	1 (12.5%)	NS
**Diagnostics**					
First brain MRI					
abnormal	7 (37%)	0	4 (80%)	3 (37.5%)	*p* = 0.044
not performed	2 (11%)	1 (16.5%)^a^	1 (20%)^a^	0	NS
SF					
pleocytosis^b^	8 (42%)	2 (33%)	4 (80%)	3 (37.5%)	NS
elevated proteins^c^	9 (47%)	1 (17%)	4 (80%)	4 (50%)	NS
diagnostic findings			HSV-1 PCR+ (4) TBE Ab+ (1)		
Ictal EEG					
focal	11 (58%)	3(50%)	3 (60%)	5 (62.5%)	NS
multifocal	3 (16%)	2 (33%)	0	1 (12.5%)	NS
generalized	1 (5%)	0	0	1 (12.5%)	NS
not available	4 (21%)	1 (17%)	2 (40%)	1 (12.5%)	NS

Footnote to Table 1. ^a^ Head computed tomography was normal. ^b^ Defined as cell count > 5 × 106/l. ^c^ Defined as > 500 mg/l in patients under 50 years and > 650 mg/l in patients over 50 years. *NS* not significant, *MRI* magnetic resonance imaging, *CSF* cerebrospinal fluid, *HSV-1 PCR* herpes simplex virus 1 polymerase chain reaction, *TBE Ab* Tick-borne encephalitis antibodies, *EEG* electroencephalogram. Data presented as number (%) and median (interquartile range)

**Table 2:** Given treatments and features of intensive care in the identified NORSE subgroups

	All patients (***n*** = 19)	FIRES (***n*** = 6)	Viral encephalitis (***n*** = 5)	Afebrile NORSE group (***n*** = 8)	FIRES vs non-FIRES
Number of antiseizure medications	4 (4-8)	9 (8-9)	3 (3-4)	4 (4-5)	*p* = 0.001
Number of anesthetics	2 (1-3)	4 (2-4)	1 (1-2)	1 (1-2)	*p* = 0.002
Antimicrobial medication	16 (84%)	6 (100%)	5 (100%)	5 (62.5%)	NS
Immunotherapy	7 (37%)	5 (83%)	1 (20%)	1 (12.5%)	0.01
STESS	4 (3-4)	4 (4-4)	3 (3-4)	4 (4-5)	NS
CBI-score	4 (2-9)	9 (7-9)	2 (1-3)	4 (2-4)	*p* < 0.001
Days in tertiary hospital	29 (15-59)	96 (78-152)	17 (15-29)	21 (10-33)	*p* = 0.017
Days in intensive care unit	16 (10-48)	69 (56-135)	12 (10-17)	11 (7-17)	*p* = 0.012
Days in anesthesia	4 (2-28)	54 (34-125)	2 (2-3)	3 (2-6)	*p* = 0.007

Footnote to Table 2. *NS* not significant, *STESS* status epilepticus severity score, *CBI-score* comlication burden index. Data presented as number (%) and median (interquartile range)

### Differences between NORSE subgroups

#### Demographics and comorbidities

The age of the FIRES patients differed from the other subgroups: median age was 21 years (IQR 19-24), whereas the median age of non-FIRES patients was 64 (IQR 55-69) years (*p* = 0.001). Gender distribution was similar in all subgroups.

None of the FIRES patients had any comorbidities, in contrast to the patients in the viral encephalitis group (3 patients; 60%) and in the afebrile NORSE group (6 patients; 75%; *p* = 0.011 FIRES vs. non-FIRES). Most common comorbidities were hypertension and diabetes mellitus. Two patients in the afebrile NORSE group had a history of breast cancer, of which the other died of metastasized breast cancer eight months after NORSE episode (possible paraneoplastic etiology).

#### Prodromal symptoms

By definition, all FIRES patients had fever before onset of SE. Four patients (80%) in the viral encephalitis group had fever as a prodromal symptom and one became febrile a day after admission to hospital. In contrast, none in the afebrile NORSE group had fever during the preceding two weeks of hospitalisation. In the FIRES group, four (67%) had respiratory infection symptoms prior to SE, in contrast to none in the other patient groups (*p* = 0.004). Half of the patients (*n* = 4) in the afebrile NORSE group had cognitive or psychiatric symptoms but none had headache, respiratory, or gastrointestinal symptoms.

#### Early diagnostic findings

All viral encephalitis patients with MRI scan taken (MRI was lacking from the patient with TBE) had abnormal findings in the first MRI scan performed 1 – 12 days after admission to hospital. In three patients, there were findings of T2-hyperintensities or hemorrhagic necrosis in temporal lobes, limbic structures and insula, and these were considered as typical for HSV encephalitis. In one patient, T2-hyperintensity and oedema located in the frontal lobe. In the afebrile NORSE group, brain MRI was performed for all patients 2 – 17 days after admission to hospital. Three of them (38%) had abnormal MRI findings (illustrated in Fig. 2). In the FIRES group, five out of six patients had MRI performed during the first three days of hospital care and all had normal findings, which differentiated the FIRES group from the viral encephalitis group (4/5 abnormal MRI, one lacking MRI). One FIRES patient died four days after admission to hospital without an MRI taken, but the head CT was normal. Two out of six (33%) FIRES patients presented T2 hyperintensities in the temporal lobes, leptomeningeal enhancement and general atrophy in the later scans.

Four patients (80%) with viral encephalitis had pleocytosis (range 6 – 330 × 10^6^/l) in the first CSF sample, while only two patients (30%) with FIRES (range 7 – 10 × 10^6^/l) and three patients (38%) in the afebrile NORSE group (range 22 – 64 × 10^6^/l) had pleocytosis. White blood cell count in the viral encephalitis group was higher but the difference was not statistically significant. Also protein was more elevated in non-FIRES groups, though the difference was not significant.

In 18 patients, SE diagnosis was based on clinical findings and in only one case it was EEG that led to the diagnosis and treatment accordingly. In five cases, emergency EEG right after the first- and second-line medications verified SE. Fifteen patients had SE finding in later EEGs. In four patients (21%), ictal EEG was not obtained since RSE diagnosis was based on clinical findings and the condition resolved with appropriate treatment, and in later EEGs ictal findings were no longer present. All patients with SRSE (ten patients) had EEG verified diagnosis. Eleven patients out of fifteen (73%) with ictal EEGs, had focal ictal findings, and no significant difference was found between subgroups. It was noteworthy that all FIRES patients whose SE continued for weeks developed multifocal seizures over the course of the disease.

#### Treatment

As a first line medication, nine patients received iv lorazepam (2 – 10 mg, mean 4 mg), five patients iv diazepam (2.5 – 10 mg, mean 6 mg) and one patient midazolam (10 mg intravenously and 1 mg buccally). In six patients, doses of the first line medication were not available in electronic patient database. Three patients received two different benzodiazepines. All patients were treated with at least three different ASMs. In two cases, focal SE resolved with ASMs, whereas in all other cases general anesthesia was required. The number of ASMs and anesthetics used was higher in the FIRES subgroup compared to other subgroups (*p* = 0.001 and *p* = 0.002, respectively). Also, immunotherapy was used significantly more often in patients with FIRES (*p* = 0.01). In three patients, the first immunotherapy was three days high-dose intravenous methylprednisolone and in two patients, intravenous immunoglobulin treatment. Immunotherapy was started median seven days after admission to hospital (IQR 6-9) in the FIRES group.

Due to SRSE, additional treatments to limit epileptic activity were given in the FIRES subgroup: vagus nerve stimulator (*n* = 2), deep brain stimulator (*n* = 1), transcranial magnetic stimulation (*n* = 1), ketogenic diet (*n* = 1), ketamine-midazolam infusion (*n* = 3), therapeutic hypothermia (*n* = 4), and magnesium infusion (*n* = 1). None of the above SRSE treatments were necessary in non-FIRES patients.

The FIRES subgroup had longer tertiary hospital stay (*p* = 0.017), intensive care treatment (*p* = 0.012), and time under anesthesia (*p* = 0.007) compared to other subgroups. The FIRES subgroup also experienced more complications than the other subgroups (*p* < 0.001). Median STESS values were equal for the FIRES and the afebrile NORSE group, as patients in the afebrile group scored points for age (four patients compared to none in the FIRES group), but had better level of consciusness (stuporous/comatose in five patients vs all in the FIRES group) and milder seizure types (non-convulsive status epilepticus in six out of eight patients compared to all in the FIRES group) than the FIRES group.

#### Outcome

Median follow-up time was 73 months (IQR 54 – 90) in the viral encephalitis group, 102 months (IQR 81 – 114) in the FIRES group, and 25 months (IQR 8 – 26) in the afebrile NORSE group. In the latter, three patients were lost from follow-up after the tertiary hospital phase, because follow-up continued in a different hospital district; for these three patients, only the mRS determined at discharge from the tertiary hospital and their date of death were available as follow-up information.

**Fig. 3:**
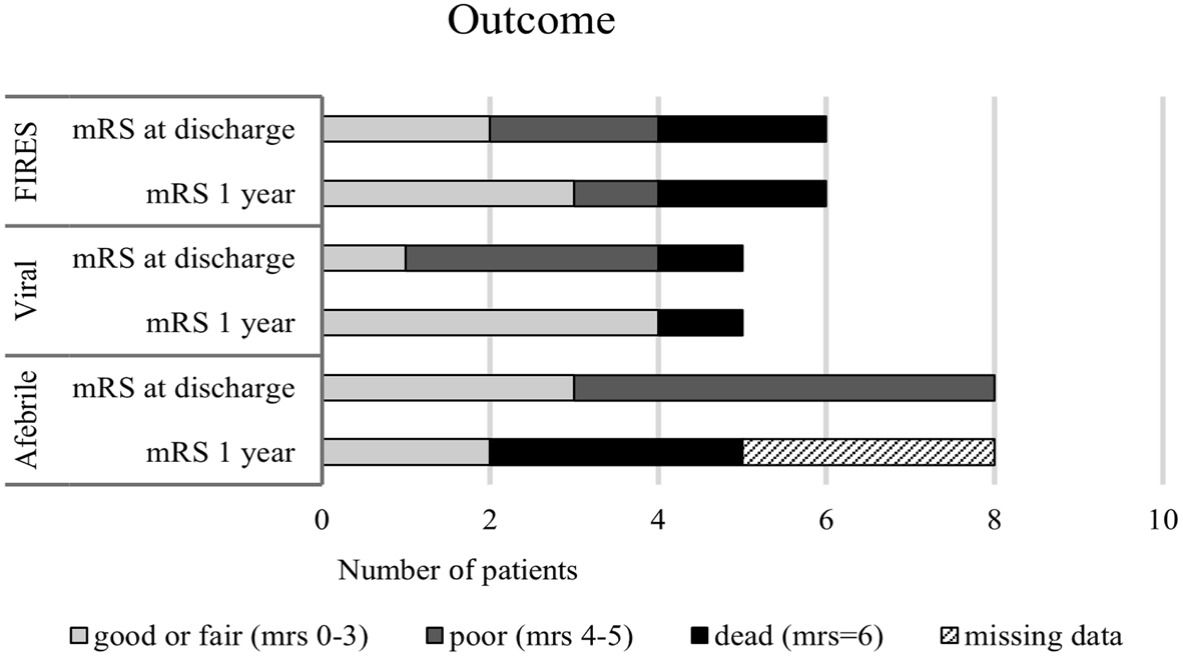
Treatment outcome in the subgroups of NORSE, given as modified Rankin Scale (mRS) at discharge and at 1-year post-hospitalisation

Outcomes of the subgroups at tertiary hospital discharge and at 1 year in terms of mRS are presented in Fig. 3 (in detail see Tables S1 – S3). All four surviving patients in the viral encephalitis group and three of four surviving patients in the FIRES group had a good or fair outcome (mRS 0-3) at 1 year after NORSE. In the afebrile NORSE group, only two of the five patients with mRS data available had a good outcome at 1 year after NORSE. The total mortality in the viral encephalitis group or in the FIRES group did not increase after the NORSE episode. In contrast, six (75%) of the patients in the afebrile NORSE group had died by the end of the follow-up in 31st December 2020.

Despite the observed differences in the clinical course and outcome between the three subgroups of NORSE, age, comorbidities, subgroup class, the duration of intensive care treatment, or complications did not explain the poor outcomes in our study sample.

In the FIRES group, all four surviving patients had epilepsy requiring polytherapy at the end of the follow-up, in contrast to only one of the four surviving patients in the viral encephalitis group. In the afebrile NORSE group, four out of five patients with follow-up data available needed sustained ASM.

### Incidence of FIRES

During the study period, the average number of adult inhabitants (≥16 years) in the Helsinki University Hospital catchment area was 1.72 million [8]. The population-based annual incidence of FIRES was thus found to be 0.03 per 100,000 persons.

## Discussion

In this retrospective study, we explored the early clinical features of NORSE to identify clinical subgroups that would help in directing the treatment in the first days of hospitalisation. Based on febrile status and identifiable etiology, we could divide patients into viral encephalitis, FIRES and afebrile NORSE subgroups. HSV-1 was the main viral agent in the viral encephalitis group, but patients in the FIRES and afebrile NORSE group remained etiologically cryptogenic. In our cohort, FIRES was clinically very distinctive due to patients’ young age, prodromal respiratory symptoms, normal first brain imaging and strikingly treatment-resistant status epilepticus.

In recent studies, interleukin antagonists like anakinra and tocilizumab have had dramatic responses in seizure cessation in FIRES patients referring to a disturbance of the innate immune system as a possible mechanism underlying this condition [5, 6]. Therefore it’s essential that the unique clinical findings of this group are taken into account already in the first days of hospitalisation and prompt initiation of interleukin antagonists is evaluated at the same time with starting first line immunotherapies.

The proportion of cryptogenic cases has varied between 52 and 77% in NORSE studies depending on the inclusion criteria and definitions used [3, 10, 11]. In our study, over two thirds of all NORSE patients remained without identified etiology, which is in line with a study of similar size and inclusion criteria [10], although infectious etiology was more common (26% vs 8%) and autoimmune encephalitis scarcer in our study (0% vs 19%). These differences can be due to different diagnostic testing practices and also small sample sizes. In our study, incomplete testing of neuronal antibodies probably has caused underestimation of autoimmune or paraneoplastic etiologies especially in the afebrile NORSE group. In the largest study of 130 NORSE patients [3], Gaspard et al. identified autoimmune etiology in 19%, paraneoplastic etiology in 18%, and infectious etiology in 8% of patients. However, the study was performed before the current NORSE definition and included only patients tested with an autoimmune or paraneoplastic panel. Thus, patients with specific etiology recognized within the first 48 hours, for example patients with viral encephalitis, were excluded, unlike in the current definition of NORSE. Similarly to the study by Gaspard et al., we also found one patient with probable Creutzfeldt-Jakob disease (CJD), which is in accordance with the growing literature on SE as a possible first manifestation of CJD [12].

From the early diagnostic findings, abnormal MRI findings have been reported in 50-80% of NORSE cases including T2 and FLAIR (Fluid-attenuated inversion recovery) changes, mainly in the neocortical and limbic areas but also in the basal ganglia and claustrum [3, 10, 13, 14]. A recent review of MRI changes in pediatric FIRES patients concluded that over 60% of patients had normal brain MRI in the initial phase of the disease and 25% had temporal lobe findings [15]. When the patients had recovered from SE to outpatient follow-up, nearly 50% had cortical atrophy and 25% mesial temporal sclerosis visible in MRI [15]. In our study, all FIRES patients had normal brain MRI in the beginning, while nearly 60% of non-FIRES patients had abnormal MRI findings. The difference may reflect an earlier imaging point in the FIRES group, but even so, only two FIRES patients developed MRI changes including T2 hyperintensities and cortical atrophy in later scans, which is in line with previous studies.

EEG findings in our study corresponded to earlier studies reporting mainly focal or multifocal seizures [3, 16–18]. It was notable that the ictal EEG changes developed to multifocal in all FIRES patients perhaps reflecting the widening of the epileptic network and structural changes in the brain followed by sustained status epilepticus.

In previous FIRES studies consisting mainly of pediatric case series, autoimmune etiology has been rare [16, 19]. CSF findings have been normal or of a mild pleocytosis [3, 20]. Neither we could find any clinically relevant neuronal antibodies in the FIRES group. Though only three out of six patients had neuronal antibodies tested, none of them had gradual development of other symptoms e.g. cognitive or psychiatric symptoms or a movement disorder, thus contradicting an autoimmune encephalitis.

In our study, thirteen (68%) of all NORSE patients had poor outcome or had died (mRS 4-6) at discharge, which is in line with previous studies [3, 10]. Median STESS values were the same for FIRES and afebrile NORSE groups, but stemmed from a more severe alteration of consciousness and worst seizure type in the young FIRES patients, whereas afebrile NORSE patients received points from age and a less severe presentation. The number of complications and duration of intensive care treatment was clearly higher in the FIRES group, but these factors did not associate with poor outcome, in contrast to previous studies [3]. Neither the clinical subgroup, age, nor comorbidities associated with poor outcome. These results might be due to small sample size but it also seems likely that young patients with FIRES also have capacity to survive and recover from the intensive care treatment. However, all FIRES patients had polytherapy-requiring epilepsy and neurocognitive problems until the end of the follow-up. Viral encephalitis patients recovered well during the first year and the majority of patients were able to discontinue the ASM during the follow-up. In the afebrile NORSE group, total mortality increased during the first year and throughout the follow-up, which is likely at least in part due to older age and higher number of comorbidities than in the other subgroups.

Studies of adult patients with NORSE have rarely distinguished the subgroup of FIRES. Historically, FIRES was defined to include only pediatric patients. In our study, the annual incidence of FIRES among adults, 0.03/100 000, was slightly lower than the reported incidence of 0.1/100 000 in the pediatric population [20]. We could not find previous studies on incidence in an adult population.

The main limitations of our study are the retrospective clinical setting and the small sample size. However, given the rarity of NORSE, our cohort is considerable and the follow-up time relatively long. The patient cohort was collected from the intensive care departments only, and therefore it is possible that some of the NORSE cases that responded to additional ASMs or short-term anesthesia at the emergency department or at emergency site were missed. Our rationale was indeed to find the treatment-resistant cases that are challenging to manage in clinical practice. It is noteworthy that the diagnosis of SE had been made based on clinical assesment rather than only EEG findings, which is however often the situation in clinical practice. Diagnosis of SRSE was always based on an SE finding in EEG. The doses of first-line medications in SE patients may often be inadequate as reported in the SENSE study [21]. Unfortunately, we were not able to verify the doses of the first line medications in all cases, but in 13 cases (68%) the verified dose was appropriate. Due to the long data collection span, autoantibody testing was in many patients insufficient in light of current good practice. However, our study aimed at identifying early clinical features of NORSE, especially FIRES, that are detectable already during the first few days of hospitalisation, a time point when even today the results of modern autoantibody panels are usually pending.

Further multicenter studies are needed to elucidate the etiology of NORSE and the role of immunotherapy especially in FIRES patients. As understanding increases, also the applicability of the term NORSE requires re-evaluation as comparing patients and treatment responses in this heterogenous population does not seem valid without a more specific subgroup analysis.

## Conclusions

FIRES was clinically very distinctive among all NORSE patients due to young age at onset, prodromal respiratory symptoms and normal first brain imaging. Of the diagnostic tests, especially normal brain imaging distinguished FIRES from patients with viral encephalitis. For afebrile, older NORSE patients with cognitive or psychiatric prodromal symptoms, paraneoplastic and neurodegenerative etiology was suspected. Given the extremely refractory nature of the SE in FIRES, usability of these clinical diagnostic findings as criteria for selecting patients for early intensive immunotherapy should be confirmed in future studies.

## Supplementary Information

**Additional file 1: Table S1.** Detailed description of the FIRES group. **Table S2. **Detailed description of the viral encephalitis group. **Table S3.** Detailed description of the afebrile NORSE group.

## Data Availability

The datasets generated and/or analyzed during the current study are not publicly available due to legislation which restricts sending or restoring the data abroad but are available from the corresponding author on reasonable request.

## Abbreviations

NORSE: New-onset refractory status epilepticus
FIRES: Febrile-infection related epileptic syndrome
ASM: Antiseizure medication
SE: Status epilepticus
RSE: Refractory status epilepticus
SRSE: Super-refractory status epilepticus
CSF: Cerebrospinal fluid
CBI: Complication burden index
STESS: Status epilepticus severity score
mRS: modified Ranking Scale
TPO: Thyroid peroxidase
VGKC: Voltage-gated kalium channel complex
LGI1: Leucine-rich glioma-inactivated 1
CASPR2: Contactin-associated protein-like 2
SOX-1: SRY-Box Transcription Factor 1
Zic4: Zinc finger protein 4
CJD: Creutzfeldt-Jakob disease
FLAIR: Fluid-attenuated inversion recovery
