# Supplementary material for: Early clinical features of new-onset refractory status epilepticus (NORSE) in adults
Source: BMC Neurol. 2022 Dec 20;22:495. doi: 10.1186/s12883-022-03028-y (PMC9764533; doi:10.1186/s12883-022-03028-y)
Supplement: Supplementary file 1 — Additional file 1: Table S1. Detailed description of the FIRES group. Table S2. Detailed description of the viral encephalitis group. Table S3. Detailed description of the afebrile NORSE group. [file 12883_2022_3028_MOESM1_ESM.docx]

**Supplemental Data**

**Table S1**. Detailed description of the FIRES group

| **Patient** | **Age** | **Sex** | **Comorbidities** | **Ictal EEG** | **Brain imaging** | **Laboratory findings** | **Additional laboratory testing with negative results** | **Immunotherapy** | **Outcome at discharge** |
| --- | --- | --- | --- | --- | --- | --- | --- | --- | --- |
| 1 | 25 | M | none | not available | normal | CSF  leuc 4 x10^6^/l  prot 423 mg/l  Serum  TPO Abs mildly elevated | CSF  IgG-index, ACE, LZM  PCR: HSV-1, HSV-2, VZV, HHV-6, Adenovirus, Enterovirus, *Mycoplasma pneumoniae*, *Borrelia burgdorferi*, *Mycobacterium tuberculosis*, *Treponema pallidum*  Ab: Cardiolipin, *Borrelia burgdorferi*, HSV-1, HSV-2, VZV, CMV, *Mycoplasma pneumoniae*, GAD  Serum  Ab: TBE, HSV-1, HSV-2, HHV6, VZV, *Mycoplasma pneumoniae*, *Chlamydia pneumoniae*, CMV, Adenovirus, Enterovirus, Influenza A and B, Parainfluenza, RSV, *Toxoplasma gondii*, HIV, ANCA, ANA, ENA, DNA, Ganglioside, TSHR, cardiolipin, GAD, Aquaporin4, VGKC, Ma2, CV2, GluR3  Other: *Treponema pallidum* hemagglutin assay, POLG1-gene, LZM, U-Aminoacid, fp-Aminoacid, U-Organic acid, IgA, IgM, IgG, IgG-Subcategories, C3, C4, lymphocyte categorization, serum and urin protein electrophoresis, lupus anticoagulant, cryoglobulin, porfyria tests, bone marrow aspiration, kidney and muscle biopsia, lymph node sample  Nasal secretion: respiratory virus antigen test* | corticosteroid + IVIG x2 | good  (mRS 2) |
| 2 | 19 | M | none | focal | normal | CSF  leuc 1 x10^6^/l  prot 405 mg/l | CSF  cytology  PCR: HSV-1, HSV-2, VZV, HHV-6, CMV, Enterovirus, *Mycoplasma pneumoniae*, *Borrelia burgdorferi*  Ab: Enterovirus, TBE, HSV-1, HSV-2, VZV, *Mycoplasma pneumoniae*, Adenovirus, Influenza A, Influenza B, Parainfluenza 1, RSV, *Treponema pallidum*, cardiolipin, NMDAR, AMPAR, GlyR, LGI1, CASPR2, GABABR, GAD, Hu, Ri, Yo, Tr(DNER), MAG, Myelin, Ma/Ta, Amfi, Aquaporin4  Flow cytometric immunophenotyping of CSF  Serum  Ab: TBE, *Borrelia burgdorferi*, HSV-1, HSV-2, HHV6, VZV, *Mycoplasma pneumoniae*, *Chlamydia pneumoniae*, EBV, CMV, Adenovirus, Enterovirus, Influenza A and B, Parainfluenza, RSV, *Toxoplasma gondii*, Coxsackie B5, HIV, ANCA, ANA, ENA, DNA, Ganglioside, TPO, cardiolipin, AMPAR, NMDAR, GlyR, VGKC, LGI1, CASPR2, GAD, Hu, Ri, Yo, Tr(DNER), MAG, Myelin, Ma/Ta, Amfi, Aquaporin4  Other: *Treponema pallidum* hemagglutin assay, POLG1-gene, IgG, IgM, IgA, C3, C4, lupus anticoagulant.  Nasal secretion: respiratory virus antigen test* | corticosteroid + IVIG x2 + plasma exchange x2 + azathioprine | poor  (mRS 4) |
| 3 | 20 | F | none | focal | only CT was performed, normal | CSF  leuc 1 x10^6^/l  prot 628 mg/l | CSF  PCR: HSV-1, HSV-2, VZV, HHV-6, *Borrelia burgdorferi*  Ab: EBV, CMV, TBE, *Borrelia burgdorferi*, HSV-1, HSV-2, VZV, *Mycoplasma pneumoniae*, *Treponema pallidum*, cardiolipin  Serum  Ab: *Borrelia burgdorferi*, TBE, HSV-1, HSV-2, HHV6, VZV, *Mycoplasma pneumoniae*, *Chlamydia pneumoniae*, CMV, EBV, Adenovirus, Enterovirus, Influenza A and B, Parainfluenza, RSV, *Toxoplasma gondii*, HIV, ANCA, ANA  Other: *Treponema pallidum* hemagglutin assay, POLG1-gene, IgA, IgM, IgG, C4, C3 | none | death  (mRS 6) |
| 4 | 18 | M | none | multifocal | normal | CSF  leuc: 0 x10^6^/l  prot 342 mg/l | CSF  oligoclonality, IgG-index, blasts, 14-3-3 marker  PCR: HSV-1, HSV-2, VZV, HHV-6, Adenovirus, Enterovirus, Parechovirus, Rhinovirus, *Borrelia burgdorferi*  Ab: TBE, Enterovirus, Coxsackie B5, Adenovirus, Influenza A, Influenza B, Parainfluenza 1, RSV, NMDAR, CRMP5, VGKC, CASPR2, LGI1, GABABR, Yo, Ma1, Ma2, Amfi, Ri, Hu, CV2, Tr(DNER), GAD65, SOX1, Zic4  Serum  Ab: TBE, HSV-1, HSV-2, HHV6, VZV, *Mycoplasma pneumoniae*, CMV, EBV, Adenovirus, CoxsackieB5, Enterovirus, Influenza A and B, Parainfluenza, RSV, HIV, TPO, ANA, ENA, DNA, cardiolipin, NMDAR, AMPAR, CRMP5, VGKC, CASPR2, GABABR, LGI1, Yo, Ma1, Ma2, Amfi, Ri, Hu, CV2, Tr(DNER), GAD65, SOX1, Zic4  Other: POLG1-gene, IgG, lupus anticoagulant, EBV-PCR, CMV-PCR | corticosteroid +IVIG + plasma exchange + allopregnalone | death  (mRS 6) |
| 5 | 28 | F | none | focal | normal | CSF  leuc 7 x10^6^/l  prot 332 mg/l  VGKC,  Sox1 and Zic4 Abs+ (mild positive)  Serum  VGKC Ab+ (mild positive) | CSF  IgG-index, oligoclonality, ACE  PCR: HSV-1, HSV-2, VZV, HHV-6, Enterovirus, *Mycoplasma pneumoniae*, *Mycobacterium tuberculosis*  Ab: TBE, *Borrelia burgdorferi*, NMDAR, AMPAR, CASPR2, LGI1, GABABR, GlyR, Yo, Ma1, Ma2, Amfi, Ri, Hu, CV2, Tr(DNER), GAD65  Serum  Ab: TBE, *Borrelia burgdorferi*, *Mycoplasma pneumoniae*, ANCA, ANA, ENA, TPO, NMDAR, AMPAR, CASPR2, LGI1, GABABR, Yo, Ma1, Ma2, Amfi, Ri, Hu, CV2, Tr(DNER), NeGAD65, SOX1, Zic4  Other: *Treponema pallidum* hemagglutin assay, POLG1-gene, IgG, IgM, IgA  Nasopharyngeal secretion: respiratory virus antigen test* and virus PCR** | corticosteroid + IVIG + plasma exchange + rituximab | poor  (mRS 4) |
| 6 | 21 | F | none | multifocal | normal | CSF  leuc 10 x10^6^/l  prot “normal” | CSF  IgG-index, oligoclonality, cytology  PCR: HSV-1, HSV-2, VZV, HHV-6, *Borrelia burgdorferi*  Ab: TBE, HSV, HSV-2, VZV, *Mycoplasma pneumoniae*, Adenovirus, Influenza A, Influenza B, Parainfluenza 1, RSV  Serum  Ab: TBE, HSV-1, HSV-2, HHV6, VZV, *Mycoplasma pneumoniae*, *Chlamydia pneumoniae*, CMV, Adenovirus, Coxsackie B5, Enterovirus, Influenza A and B, Parainfluenza, RSV, HIV, StrDNA, TPO, ANCA, ANA, DNA, ANAAb-AIgA, cardiolipin, VGKC, NMDAR, Amfi, GAD, neuronal antibody immunohistochemistry, (a year later, additional tests from serum: NMDAR, AMPAR, VGKC, LGI1, CASPR2, GABABR, GlyR, Hu, Ri, Yo, Tr(DNER), MAG, Myelin, Ma2, Amfi, Aquaporin4)  Other: *Treponema pallidum* hemagglutin assay, CMV-PCR, *Mycobacterium tuberculosis* IFN-ɣ, POLG1-gene, LZM, ADA, ACE, IgM, IgG, IgG4, PC3, PC4, P-IL2R, serum protein electrophoresis, lupus anticoagulant, cryoglobulin, CA12-5, serum thymidine kinase | corticosteroid x2 + IVIG + plasma exchange + azathioprine | good  (mRS 3) |

*Adeno, Boca, Human metapneumovirus, Influenza A, Influenza B, Corona, Picorna, Parainfluenza 1,2,3,4, Rhino, RSV

**Adeno, Influenza A, Influenza B, Parainfluenza 1, 2,3, RSV

**Table S2.** Detailed description of the viral encephalitis group

| **Patient** | **Age** | **Sex** | **Comorbidities** | **Ictal EEG** | **Brain imaging** | **Laboratory findings** | **Additional testing with negative results** | **Immunotherapy** | **Outcome at discharge** |
| --- | --- | --- | --- | --- | --- | --- | --- | --- | --- |
| 7 | 64 | F | hypertension,  diabetes mellitus type 2 | focal | only CT was performed, normal | CSF  leuc 112 x10^6^/l  prot 787 mg/l  TBE Ab +  Serum  TBE Ab + | CSF  PCR: HSV-1, HSV-2, VZV, HHV-6, *Borrelia burgdorferi*  Ab: *Borrelia burgdorferi*  Serum  Ab: *Borrelia burgdorferi*, *Mycoplasma pneumoniae* | none | poor  (mRS 4) |
| 8 | 55 | F | none | focal | T2-hyperintensity in the right frontal lobe | CSF  leuc 2 x10^6^/l  prot 291 mg/l  HSV-1 PCR + | CSF  Oligoclonality  PCR: HSV-2, VZV, HHV-6  Ab: *Borrelia burgdorferi*  Serum  Ab: TBE, *Borrelia burgdorferi*, ANCA, ANA, ENA, DNA  Nasal secretion: virus PCR* | none | death (mRS 6) |
| 9 | 46 | M | none | not available | hemorrhagic necrosis and oedema in right temporal lobe and insula | CSF  leuc 330 x10^6^/l  prot 890 mg/l  HSV-1 PCR + | CSF  PCR: HSV-2, VZV, HHV-6, *Borrelia burgdorferi*, *Treponema pallidum*  Ab: *Mycoplasma pneumoniae*  Serum  Ab: TBE, *Borrelia burgdorferi*, *Mycoplasma pneumoniae*, Adenovirus, *Chlamydia pneumoniae*, Enterovirus, Influenza A and B, Parainfluenza, RSV, *Toxoplasma gondii*, HIV  Nasal secretion: Influenza A and B antigen | none | good  (mRS 1) |
| 10 | 64 | M | diabetes mellitus type 2 | focal | T2-hyperintensity in left temporal lobe, insular cortex, hippocampus and pulvinar thalamus and bilaterally in frontal lobes | CSF  leuc 10 x10^6^/l  prot 697 mg/l  HSV-1 PCR + | CSF  PCR: HSV-2, VZV, HHV-6, Enterovirus  Ab: HSV-1, HSV-2, VZV, *Mycoplasma pneumoniae*  Serum  Ab: Adenovirus, Influenza A and B, RSV, *Chlamydia pneumoniae*, Enterovirus, *Mycoplasma pneumoniae*, Parainfluenza, *Toxoplasma gondii*, ANCA, ANA  Nasal secretion: respiratory virus antigen test** | none | poor  (mRS 4) |
| 11 | 65 | M | depression,  vitiligo | not available | T2-hyperintensity in insular cortex, cingulum, right thalamus, and hippocampus | CSF  leuc 6 x10^6^/l  prot 773 mg/l  HSV-1 PCR+ | CSF  13-4-4 marker, Fostau, Tau, Bm42, IgG-Index  PCR: HSV-2, VZV, HHV-6, *Mycoplasma pneumoniae*, *Chlamydia pneumoniae*  Ab: *Borrelia burgdorferi*, HSV 1 and 2, NMDAR, AMPAR, CASP2R2, GABABR, LGI1, Yo, Ma1, Ma2, Amfi, Ri, Hu, CV2, Tr(DNER), GAD65, SOX1, Zic4  Serum  Ab: TBE, HSV-1, HSV-2, HHV6, VZV, *Mycoplasma pneumoniae*, NMDAR, AMPAR, VGKC, CASPR2, GABABR, LGI1, Yo, Ma1, Ma2, Amfi, Ri, Hu, CV2, Tr(DNER), GAD65, SOX1, Zic4, GAD | corticosteroid + IVIG | poor  (mRS 5) |

* Adeno, Boca, Human metapneumovirus, Influenza A, Influenza B, Corona, Picorna, Parainfluenza 1,2,3,4, Rhino, RSV

**Adeno, Influenza A, Influenza B, Parainfluenza 1, 2,3, RSV

**Table S3.** Detailed description of the afebrile NORSE group

| **Patient** | **Age** | **Sex** | **Comorbidities** | **Ictal EEG** | **Brain imaging** | **Laboratory findings** | **Additional laboratory testing with negative results** | **Immunotaherapy** | **Outcome at discharge** |
| --- | --- | --- | --- | --- | --- | --- | --- | --- | --- |
| 12 | 48 | F | none | focal | T2-hyperintensity bilaterally in temporal lobes, amygdala, hippocampus, and thalamus | CSF  leuc 0 x10^6^/l  prot 482 mg/l | CSF  PCR: HSV-1, HSV-2, VZV, HHV-6  Ab: *Borrelia burgdorferi*  Serum  Ab: HSV-1, HSV-2, HHV6, VZV, *Mycoplasma pneumoniae*, NMDAR, VGKC, LGI1 | none | good  (mRS 2) |
| 13^1^ | 63 | F | chronic reactive arthritis (HLA-B27 positive),  13 years earlier mastectomy, radiation therapy and cytostatic treatment of gradus 3 ductal mamma ca | focal | normal | CSF  leuc 0 x10^6^/l  prot 472 mg/l | CSF  PCR: HSV-1, HSV-2, VZV, HHV-6 | none | poor  (mRS 5) |
| 14^2^ | 64 | M | asthma | focal | normal | CSF  leuc 69 x10^6^/l  prot 666 mg/l | CSF  cytology  PCR: HSV-1, HSV-2, VZV, HHV-6, Enterovirus, *Mycoplasma pneumoniae*, Influenza-A  Serum  Ab: TBE, *Borrelia burgdorferi*, HSV-1, HSV-2, HHV6, VZV, *Mycoplasma pneumoniae*, HIV, ANCA, ANA, ENA, DNA, ANA  Other: TPHA, IgA, IgG, IgM, C4 ja C3, serum and urin protein electrophoresis, cryoglobulin  Nasal secretion  Influenza A and *Mycoplasma pneumoniae* PCR, respiratory virus antigen test* | none | poor  (mRS 5) |
| 15^3^ | 69 | F | hypertension | not available | normal | CSF  leuc 1 x10^6^/l  prot 539 mg/l | CSF  oligoclonality, IgG-index  PCR: HSV-1, HSV-2, VZV, HHV-6, Enterovirus, *Treponema pallidum*  Ab: *Borrelia burgdorferi*, HSV-1, HSV-2, VZV, *Mycoplasma pneumoniae*  Serum  Ab: HSV-1, HSV-2, HHV6, VZV, *Mycoplasma pneumoniae* | none | good  (mRS 2) |
| 16^4^ | 72 | F | arthrosis | focal | high signal in DWI sequences in the head of the right caudatus nucleus and in the frontal part of the right putamen | CSF  leuc 2 x10^6^/l  prot 564 mg/l  CSF 14-3-3 marker positive after discharge | CSF  ACE, LZM, Fostau, Tau, Bm42, 13-4-4  PCR: HSV-1, HSV-2, VZV, HHV-6, *Treponema pallidum*  Ab: *Borrelia burgdorferi*, *Mycoplasma pneumoniae*, HSV-1, HSV-2, VZV  Serum  Ab: HSV-1, HSV-2, VZV, *Mycoplasma pneumoniae*  S-TPO, NMDAR, AMPAR, VGKC, GABABR, GlyR, Yo, Ma1, Ma2, Amfi, Ri, Hu, CV2, Tr(DNER), GAD65, SOX1, Zic4  S-ACE | none | poor  (mRS 5) |
| 17 | 71 | F | mastectomy and radiation therapy of gradus 1 ductal mamma ca 11 years earlier,  hysterectomy and ovariectomy due to benign tumors | multifocal | bilateral frontal, temporal and parietal T2-hyperintensities cortically and subcortically and mildly in periventricular area | CSF  leuc 16 x10^6^/l  prot 687 mg/l | CSF  oligoclonality  PCR: HSV-1, HSV-2, VZV, HHV-6, *Borrelia burgdorferi*, Enterovirus  Ab: TBE, *Borrelia burgdorferi*  brain biopsia; pyknotic, acidofilic neurons intracortically, subcortical oedema, wide gliosis  Serum  Ab: *Borrelia burgdorferi*, HIV, cardiolipin.  Other: *Treponema pallidum* hemagglutination assay | none | poor  (mRS 5) |
| 18 | 79 | F | hypertension,  diabetes mellitus type 2 | focal | normal | CSF  leuc 2 x10^6^/l  prot 327 mg/l | CSF  PCR: HSV-1, HSV-2, VZV, HHV-6  Ab: *Borrelia burgdorferi*  Serum  Ab: TBE, *Borrelia burgdorferi*, HIV | none | good  (mRS 2) |
| 19 | 45 | M | none | generalized | normal | CSF  leuc 22 x10^6^/l  prot 855 mg/l | CSF  oligoclonality, IgG-index,  CSF PCR: HSV-1, HSV-2, VZV, HHV-6, *Treponema pallidum*  Ab: GlyR, AMPAR  Serum  Ab: TBE, *Borrelia burgdorferi*, HSV-1, HSV-2, HHV6, VZV, *Mycoplasma pneumoniae*, *Chlamydia pneumoniae*, CMV, EBV, Adenovirus, Enterovirus, Influenza A and B, Parainfluenza, RSV, *Toxoplasma gondii*, HIV, Orthohantavirus, Japanese encephalitis virus, *Coxiella burnetii*, NMDAR, VGKC, GlyR, AMPAR, GABABR, GAD  Other: POLG1-gene, *Treponema pallidum* hemagglutination assay  Nasal secretion: respiratory virus antigen test, Influenza A PCR | corticosteroid + IVIG | poor  (mRS 5) |

*Adeno, Boca, Human metapneumovirus, Influenza A, Influenza B, Corona, Picorna, Parainfluenza 1,2,3,4, Rhino, RSV

^1^ possible paraneoplastic syndrome: died of metastazied breast cancer 8 months after NORSE

^2^ possible paraneoplastic syndrome: died of metastazied lung cancer 28 months after NORSE

^3^ possible frontotemporal dementia

^4^ possible prion disease
